# Different Role of CSF MMPs in Amyloid Pathology and Cerebrovascular Pathology

**DOI:** 10.1002/alz70856_098315

**Published:** 2025-12-24

**Authors:** Feng Gao, Yaxi Zhan, Yong Shen

**Affiliations:** ^1^ Department of Neurology, Institute on Aging and Brain Disorders, The First Affiliated Hospital of USTC, Division of Life Sciences and Medicine, University of Science and Technology of China, Hefei, Anhui, China; ^2^ University of Science and Technology of China, Hefei, 233026, China; ^3^ University of Science and Technology of China, Neurology Department, Institute on Aging and Brain Disorders, Hefei, China

## Abstract

**Background:**

Amyloid and cerebrovascular pathologies play critical role in the pathogenesis of Alzheimer's disease (AD). Matrix metalloproteinases (MMPs) is known to degrade Aβ peptides, while they also contribute to cerebrovascular pathology. Presently, the precise roles of MMPs in AD remain unclear. Therefore, we investigated relationships between CSF levels of MMPs and amyloid and cerebrovascular pathologies.

**Method:**

A total of 299 participants were included from the CANDI cohort, including 50 cognitively unimpaired (CU) individuals, 69 patients with MCI, 137 AD patients, and 43 non‐ADD patients. MMP2, MMP9, Aβ38, Aβ40 and Aβ42 were measured in the CSF. Participants underwent structural MRI and AV45 PET scan. And fluid biomarkers (Qalb and CSF PDGFRβ) were used to assess cerebrovascular pathologies.

**Result:**

CSF MMP2 levels in MCI and AD patients were significantly lower compared to CU individuals and non‐ADD patients. Decreased CSF MMP2 levels, but not MMP9 were associated with lower levels of CSF Aβ peptides (Aβ38, Aβ40, Aβ42) and higher Aβ PET SUVR. Notably, CSF MMP2 was positively associated with multiple cortical regions within the AD continuum, but not in the non‐ADD continuum. Meanwhile, we observed positive correlations between CSF MMP2 and MMP9 with cerebrovascular pathologies, including WMH volume, CMB, lacunes, PVS, and Qalb. Additionally, lower CSF MMP2 was associated with longitudinal brain atrophy.

**Conclusion:**

This study suggested the involvement of MMPs in the cerebrovascular damage and the protective role of MMP2 in amyloid pathology, which highlight that the clinical application of MMP inhibitors should consider the protective effect of MMP2 on amyloid pathology.

